# Applying torque to the *Escherichia coli* flagellar motor using magnetic tweezers

**DOI:** 10.1038/srep43285

**Published:** 2017-03-07

**Authors:** Maarten M. van Oene, Laura E. Dickinson, Bronwen Cross, Francesco Pedaci, Jan Lipfert, Nynke H. Dekker

**Affiliations:** 1 Department of Bionanoscience, Kavli Institute of Nanoscience, Delft University of Technology, van der Maasweg 9, 2629 HZ Delft, The Netherlands; 2 Department of Physics, Nanosystems Initiative Munich, and Center for NanoScience, LMU Munich, Amalienstrasse 54, 80799 Munich, Germany

## Abstract

The bacterial flagellar motor of *Escherichia coli* is a nanoscale rotary engine essential for bacterial propulsion. Studies on the power output of single motors rely on the measurement of motor torque and rotation under external load. Here, we investigate the use of magnetic tweezers, which in principle allow the application and active control of a calibrated load torque, to study single flagellar motors in *Escherichia coli*. We manipulate the external load on the motor by adjusting the magnetic field experienced by a magnetic bead linked to the motor, and we probe the motor’s response. A simple model describes the average motor speed over the entire range of applied fields. We extract the motor torque at stall and find it to be similar to the motor torque at drag-limited speed. In addition, use of the magnetic tweezers allows us to force motor rotation in both forward and backward directions. We monitor the motor’s performance before and after periods of forced rotation and observe no destructive effects on the motor. Our experiments show how magnetic tweezers can provide active and fast control of the external load while also exposing remaining challenges in calibration. Through their non-invasive character and straightforward parallelization, magnetic tweezers provide an attractive platform to study nanoscale rotary motors at the single-motor level.

The bacterial flagellar motor (BFM) is a rotary motor at the heart of propulsion in many species of swimming bacteria^1^. In *Escherichia coli*, cells usually have 2–6 BFMs^2^,^3^, where each motor rotates a helical flagellar filament connected to the basal body by a flagellar hook. The flagellar motors are embedded in the cell membrane, and each consists of a rotor surrounded by multiple stators. The rotor rotates with respect to the cell body and drives the flagellar filament, whereas the stators anchor to the cell wall and exert a torque on the rotor exploiting a transmembrane electrochemical gradient^4^. When all motors rotate counter clockwise, the filaments form a flagellar bundle at a posterior end of the cell body^5^ to propel the cell at speeds of roughly^6^ 30 μm/s. In contrast, when one of the motors rotates clockwise, the flagellar bundle unbundles causing erratic cell motion^6^,^7^. By alternating between directed and erratic cell motion, the bacterium scans its environment for nutrients and other chemotactic stimuli, and, if necessary, responds by adjusting its cell motion. Thus BFMs are a key component in the foraging and survival mechanisms of swimming bacteria.

A number of techniques have been used to quantitatively probe the behaviour of individual BFMs by determining the motor’s torque-speed curve, which is analogous to force-velocity curves used to characterize linear motors^8^. In particular, torque-speed curves of BFMs have been profiled using the tethered-cell-body assay and the tethered-bead assay. In the tethered-cell-body assay^9^,^10^,^11^, the cell is tethered to a surface by its flagellum, and hence motor rotation results in rotation of the cell body. Conversely, in the tethered-bead assay^12^,^13^, the cell body is fixed to a surface, and a bead is attached to the hook or flagellar stub such that motor rotation results in rotation of the bead. In both techniques, the load torque on the motor is based on drag, which limits torque control to either adjusting the viscosity of the surrounding medium or using beads of different sizes. Extensions to these techniques include electrorotation^14^,^15^,^16^ and the use of lever-arm configurations in optical tweezers^17^,^18^,^19^,^20^, which provide more active control over the torque in terms of magnitude and direction. Both electrorotation and optical tweezers assays, however, are difficult to calibrate, require challenging experimental preparations, and/or tend to damage the probed cells due to the presence of high electrical fields.

Here, we present a magnetic tweezers assay to study the flagellar motor. The assay provides complete and active control over the external torque applied to the motor. In our assay, cells are affixed to a flow cell surface and beads are attached to the hook of the flagellar motors, similar to the tethered-bead assay. However, in our assay, the use of magnetic beads enables precise control of the applied torque to the BFM, as our magnetic tweezers instrument allows manipulation of the external magnetic field experienced by the bead (Fig. 1A). In general, application of an external magnetic field 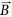 gives rise to a magnetic torque (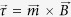), because the magnetic dipole moment 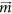 inside the bead tends to align with the external field (Fig. 1B)^21^. Magnetic tweezers have several advantages over previous techniques^22^. In particular, magnetic tweezers i) enable continuous control over torque and orientation of the magnetic particle, allowing us to brake and stall the motor and enforce rotation; ii) enable long *in vivo* measurements (several hours) that do not damage the cells due to the use of innocuous magnetic fields; and iii) facilitate multiplexing, i.e., they enable parallel measurements on multiple individual BFMs simultaneously. Overall, these properties render magnetic tweezers a powerful tool to study the flagellar motor. Here, we describe our magnetic tweezers assay and demonstrate its application to investigate the response of the BFM to different load torques and imposed backwards rotation.

## Results

**Figure 1:**
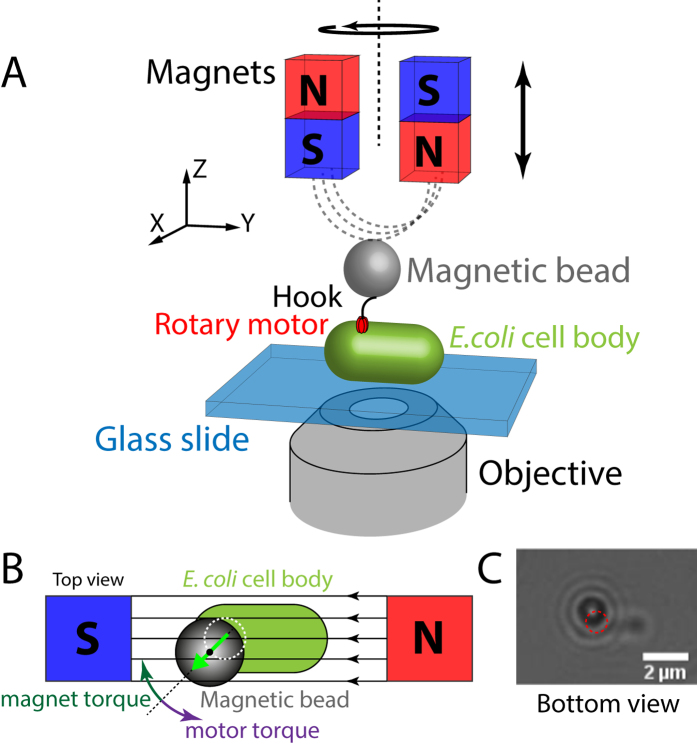
Magnetic tweezers assay for studying the *E. coli* flagellar motor. (**A**) Schematic of the experimental configuration (not to scale). The magnetic bead (grey) is connected to the flagellar motor (red) of *E. coli* by its flexible hook (black). The *E. coli* cell body (green) is fixed to a glass slide (blue). Two permanent magnets generate the external magnetic field (dashed grey lines). The objective images the sample plane onto a camera. (**B**) Top view of the experimental configuration (not to scale). The motor rotates the bead counter clockwise (purple arrow, the motor torque vector points out of the plane), while the magnet torque tends to align the magnetic bead with the field lines. At this orientation of the bead, the magnet torque vector points into the plane and pull the bead in the clockwise direction. (**C**) Camera image (bottom view) of a magnetic bead (black circle with white centre and diffraction rings) rotated by an *E. coli* cell (scale bar 2 μm). The bead traces out a circle indicated by the red dashed line.

To probe the response of the BFM to externally applied torques, we attached *E. coli* cells to a flow cell surface and tethered magnetic beads to the flagellar hooks (Fig. 1A). In our setup, two permanent magnets generate a magnetic field (Fig. 1B)^23^, which enables us to control the field strength^24^, and hence the magnetic torque, by changing the distance of the magnets from the sample. If the magnetic torque is larger than the motor torque, we can also control the orientation of the bead and, indirectly, the orientation of the motor by rotating the magnets. The position of the bead is monitored in real time by video microscopy (Fig. 1C).

**Figure 2:**
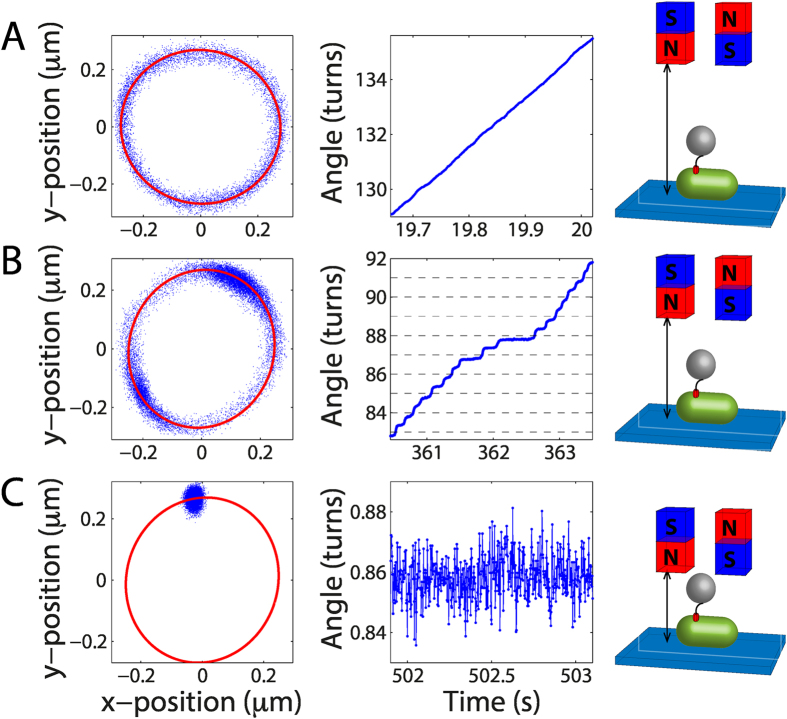
Different regimes of motor rotation depending on magnet distance. Each data point (blue) corresponds to the tracked position of the bead in a single image. Positions of the bead in (*x, y*) (left) and a zoom-in on the angular position vs. time (middle) under the following conditions: (**A**) the magnets are far from the sample (field of 1 mT; schematic on the right) and the magnetic torque is negligible; (**B**) the magnets are at an intermediate distance (field of 9 mT; schematic on the right) and the magnetic torque brakes the motor; and (**C**) the magnets are close to the sample (field of 20 mT; schematic on the right) and the magnetic torque stalls the motor. In (**A**), the full trace is approximately 15 s, and in (**B**) and (**C**), the full traces are approximately 30 s. In (**A**) and (**B**), the red lines are ellipses that have been fit to the data. In (**C**), the red line is a previous ellipse fit to rotation data of the same motor (the fit obtained in (**B**)).

To illustrate the capabilities of our assay, we gradually reduce the average speed of the motors by increasing the external magnetic field strength until the motors stall. For each recorded frame (Fig. 1C), we determine the bead position (Fig. 2, left) and extract the corresponding angular position (Fig. 2, middle; Supplement S6). When the magnets are far from the sample (Fig. 2A), the magnetic torque is close to zero and only the viscous drag torque on the bead opposes the motor. In this scenario, the bead traces out an ellipse (Fig. 2A, left; see Materials and Methods for the geometrical basis underlying the elliptical trajectory) and the angular position of the bead increases linearly in time indicating a constant rotation speed (17.5 Hz in Fig. 2A, middle). The magnetic torque becomes apparent as the magnets approach the sample plane. If the motor torque remains high enough to overcome the opposing magnetic torque, the motor rotates the bead over full revolutions (Fig. 2B) at speeds dependent on the angular position of the bead. The rotational speeds observed during one revolution reflect the potential of the bead within the magnetic field. The magnetic torque both opposes and assists the motor twice during one revolution (Fig. 2B, middle). If the magnetic field is further increased, the motor torque is eventually no longer sufficient to overcome the magnetic torque and the motor stalls (Fig. 2C). We note that complete stalling of the motor is theoretically not possible with drag-based methods such as the tethered-bead assay: the rotation speed approaches zero merely asymptotically as the drag coefficient increases to infinity. The ability to reach absolute stall illustrates one of the advantages of our magnetic tweezers assay.

### Average rotation speed versus magnetic field

**Figure 3:**
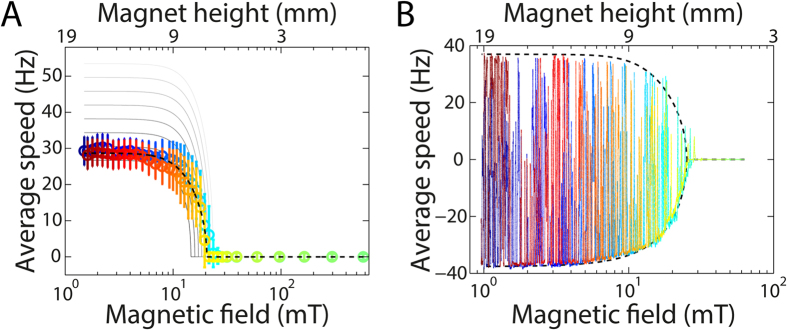
Average motor speed as a function of external magnetic field. Colour coding (from blue to red) indicates chronological order of measurements, i.e., the magnetic field strength first increases and then decreases. (**A**) Non-switching motor (MTB32 + ΔCheY), gap between magnets 1 mm. The magnets move in discrete steps. The speed is averaged over a full trace (usually 15 s or 30 s) recorded at a distinct magnet distance (top axis) and field (bottom axis). Positive speeds correspond to counter clockwise rotation. The error bars indicate the standard deviation. The grey lines are co-plotted deterministic approximations for different values of the motor torque, increasing from black to white from 700 pN·nm to 1400 pN·nm. (**B**) Switching motor (MTB47), gap between magnets 2 mm. The magnets move continuously. The speed is low-pass filtered by convolution in the time domain with a one-second-boxcar function. For clarity of presentation, no error bars are included in (**B**). Both experiments took approximately 22 minutes. Positive speeds correspond to counter clockwise rotation whilst negative speeds correspond to clockwise rotation. The dashed lines in both panels are fits to the data with fitting parameters (A) NV = 7.9·10^−3^ μm^3^, C = 0.35 kJ/m^3^, τ_motor_ = 766 pN·nm and (**B**) NV = 7.8·10^−3^ μm^3^, C = 0.43 kJ/m^3^, τ_motor_ = 988 pN·nm.

As a quantitative indicator of motor performance, we extract the average speed of rotation from the angular signal. The average speed as a function of magnetic field strength was obtained for two different strains: a non-switching strain, in which the motors rotate exclusively counter clockwise (Fig. 3A), and a switching strain, in which the motors switch rotation direction on the time scale of our experiment (Fig. 3B). At low applied external magnetic fields, the average rotation speed 〈*ω*〉 of the motor is maximal and saturates at fields below ≈5 mT. The mean speed decreases when the external field is increased above ≈5 mT. At even higher fields, but generally still below 30 mT, the motor eventually stalls, which corresponds to 〈*ω*〉 = 0. For the switching strain, the magnitude of the speed is symmetric in the clockwise and counter clockwise directions. This observation is consistent with previous reports^25^ that demonstrated symmetry in speeds up to approximately 50 Hz. We now address the low field limit, the high field limit, and the intermediate regime in turn.

### Average speed in the low field limit

In the low field limit (<5 mT), the magnetic torque becomes negligible and our assay is equivalent to a simple tethered-bead assay. Because the thermal torque averages to zero on the time scale of our speed measurements (≥1 s), the motor torque is directly related to the drag coefficient and the speed (ignoring internal friction in the motor and drag on the hook):


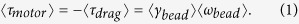


To calculate the motor torque, we measure the angular speed *ω*_*bead*_ and estimate the drag coefficient *γ*_*bead*_ based on the bead radius, bead trajectory, and viscosity of the surrounding medium (Equation S34 of ref. 21). Surface effects between the bead and glass slide or cell body are neglected for simplicity. In this low field, drag-limited regime, we find a motor speed of 30.5 ± 6.9 Hz (mean ± standard deviation from 18 cells) corresponding to a motor torque of 874 ± 206 pN·nm. The speed is in good agreement with values obtained in previous tethered-bead assays^26^ using one-micron beads, ≈32 Hz.

### Stall torque in the high field limit

In the high field limit (>30 mT), the magnetic torque balances the motor torque and the motor stalls, i.e. 〈*ω*〉 = 0, therefore:


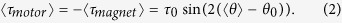


**Figure 4:**
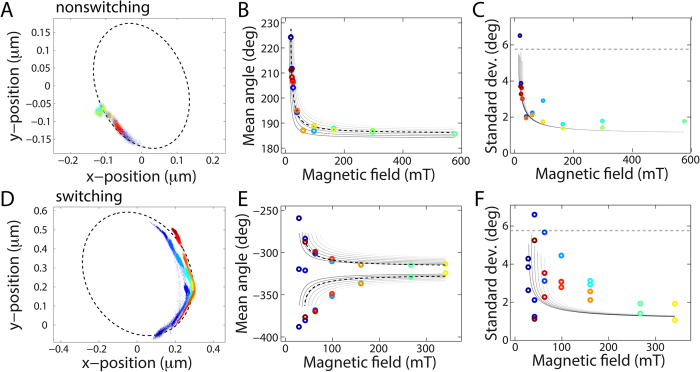
Motor stall. Colour coding (from blue to red) indicates the chronological order of the measurements, i.e., the magnet-to-sample distance first decreases and then increases. A non-switching motor (MTB32 + ΔCheY) with a 1 mm gap between the magnets (**A–C**) and a switching motor (MTB47) with a 2 mm gap between the magnets (**D–F**) stall at fields above ≈25 mT. (**A,D**) Bead positions during stall. The dashed lines are ellipse fits to rotation data of the same motor. (**B,E**) Mean angular position at different magnetic field strengths. Increased magnetic torque on clockwise (counter clockwise) motion results in decreases (increases) in the mean angle. We note that in panel (E) there appear two stall points at low field near *θ*_*0*_ = −320°, approximately midway between the two stalling positions. Possibly, the bead nonspecifically adhered here while moving between its two stable stall positions. The grey lines are co-plotted deterministic approximations (Equation 3), differing only in the motor torque. The motor torque increases from black to white from (**B**) *τ*_*motor*_ = 200 pN·nm to 800 pN·nm with *θ*_*0*_ = 183° and (**E**) *τ*_*motor*_ = ±800 pN·nm to ±2000 pN·nm with *θ*_*0*_ = −320°. The black dashed lines are fits to the data with fitted parameters (**B**) *τ*_*motor*_ = 390 pN·nm and *θ*_*0*_ = 184° and (**E**) *τ*_*motor*_ = ±1168 pN·nm and *θ*_*0*_ = −323°. (**C,F**) Standard deviation in angular position at different magnetic field strengths. The grey lines are co-plots of Equation 4 with *κ*_*hook*_ = 400 pN·nm/rad^17^,^18^, *κ*_*trap*_ is based on previous results^21^, and *τ*_*motor*_ as in (**B**) and (**E**), respectively. The dotted lines indicate the fluctuations under minimal stiffness, only due to the hook for *κ*_*hook*_ = 400 pN·nm/rad.

On the right hand side, we have introduced an analytical approximation for the magnetic torque (Supplement S2), where the parameter *τ*_*0*_ solely reflects the strength of the magnetic trap, whereas (*θ* − *θ*_*0*_), the angular shift between the anisotropy axis of the bead and the field, is determined by the interplay between motor torque and magnetic torque. To calculate the stall torque, we determine the angular position *θ* and evaluate its mean 〈*θ*〉 and variance 〈*θ*^2^〉 (Fig. 4), which contain information about both the magnetic trap strength *τ*_*0*_ and the BFM torque *τ*_*motor*_. We first evaluate 〈*θ*〉 and 〈*θ*^2^〉 before calculating the stall torque.

The mean orientation of the bead 〈*θ*〉 changes with the magnitude of the applied external field (Fig. 4). The stall position of the bead moves along the ellipse as the field is increased and then decreased again (going from blue to red in Fig. 4A and D). The mean bead orientation 〈*θ*〉 provides a quantitative visualization of this trend (Fig. 4B and E), and its value is predicted by rearranging Equation 2:


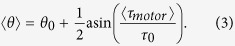


In the stall experiments, the orientation of the external magnetic field remains fixed. The exact centre of the rotational trap, *θ*_*0*_, may be offset from the orientation of the magnetic field lines due to the orientation of the magnetic anisotropy axis inside the bead. As the orientation of the bead’s magnetic anisotropy axis is unknown *a priori*, we treat *θ*_*0*_ as a fitting parameter. For the non-switching strain, we observe a single stalling orientation, which levels off at high fields (Fig. 4B) approaching *θ*_*0*_. For the switching strain, the system exhibits two stalling orientations: one for each direction of rotation. When such a motor is stalled, the magnetic torque and motor torque balance. If the motor then switches rotation direction, the motor no longer opposes the magnetic torque and instead acts in the same direction as the magnetic torque resulting in bead movement. After the bead passes alignment, i.e., after passing through (*θ* − *θ*_*0*_) = 0, the magnetic torque once again opposes the motor torque. The motor continues to displace the bead until the motor stalls at an orientation that is opposite to the previous stall position with respect to *θ*_*0*_ (Fig. 4E only displays the stall positions but not the trajectories between the stall positions). For both strains, the observations are in approximate agreement with predictions (grey lines in Fig. 4B and E; Supplement S5).

The spread in the angular position of the bead, which is quantitatively visualized by its variance 〈*θ*^2^〉 (Fig. 4C and F), also changes as the magnetic field is increased and then decreased (going from blue to red in Fig. 4A and D). In the harmonic approximation, the variance in bead position is given by the equipartition theorem:


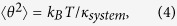


where *k*_*B*_ is the Boltzmann constant and *T* is the temperature. In our motor-bead-magnetic trap system, the stiffness is the sum of two contributions: the stiffness of the magnetic trap, *κ*_*trap*_, and the stiffness of the hook, *κ*_*hook*_, which act in parallel such that *κ*_*system*_ = *κ*_*trap*_ + *κ*_*hook*_ (Supplement S8). The trap stiffness is given by:


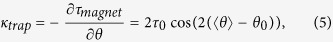


which may be rewritten using Equation 3 as:


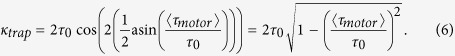


As the magnetic field strength *B* increases, the bead orientation approaches alignment, i.e., (*θ* − *θ*_*0*_) → 0, and the trap stiffness approaches its maximum value, i.e., *κ*_*trap*_*(B)* → 2*τ*_*0*_(*B*). We analyse the fluctuations in angular position (Fig. 4C and F). The fluctuations are expected to decrease with increasing field strength (Supplement S5). For both strains, the standard deviation in angle decreased with increasing field strength (dashed lines Fig. 4C and F). However, it should be noted that the range in standard deviation is large; values at the same magnet height may vary by a factor of 2–3.

The stall torque can be calculated using either the mean angular position 〈*θ*〉 (Equation 3) or the variance 〈*θ*^2^〉 (Equation 4 with the appropriate terms). We opted to use Equation 3, because the data for the mean bead orientation (Fig. 4B and E) are less noisy than the data collected for the angular fluctuations (Fig. 4C and F). We find *τ*_*0*_(*B*) by fixing the known magnetic parameters, namely the saturation magnetization *M*_*sat*_, the characteristic field *B*_*0*_, the anisotropy constant *C*, and the effective volume of superparamagnetic nanoparticles *NV*, to literature values^21^,^24^. Then we fit Equation 3 to the data (black dashed lines in Fig. 4B and E) with fitting parameters *θ*_*0*_ and τ_motor_, assuming that τ_motor_ remains constant during stall and is equal for opposite directions of rotation^25^. For the stall torque, we obtain τ_stall_ = 444 ± 366 pN·nm (mean ± standard deviation, 7 cells). Although this measured stall torque is lower than our drag-based measurement in the low-field limit, the two agree within experimental error, as expected in this high load regime. We note that the published literature values for the stall torque span a large range. The result of our measurement is consistent with several previously reported values^20^. However, its numerical value is lower than a separate direct stall torque measurement of 4500 pN·nm^19^ which, however, appears to be an outlier in the published literature^27^. Our measurement also falls below indirect, extrapolated estimates of 1260 pN·nm^28^. These estimates were measured in a resurrection experiment at full induction of the torque-generating stators; as such, they could serve as an upper limit. In our measurements, the exact number of stators per motor is unknown. The expression of stators in our measurements, an indicator for the number of stators present in the cell membrane, is either genomic or from a plasmid (Materials and Methods).

### Average speed at intermediate fields

After evaluating the low and high field limits, we focus on the intermediate regime, where the magnetic torque will brake, but not stall, the motor and the average rotational motor speed is a function of magnetic field strength (Fig. 3). For both strains, the average rotational speed of the bead varied between zero and its drag-limited speed (i.e., the speed in a tethered-bead assay) depending on the distance between the magnet and the sample. To obtain a quantitative understanding of motor behaviour, we simulated the rotational motion of a magnetic bead in an external field under the influence of thermal fluctuations, viscous drag, and a constant motor torque (Supplement S5). In addition, we fit the data to a simple model that describes the magnetic bead as a particle in a tilted periodic potential (Supplement S3).

The deterministic approximations (Supplement S3) are in qualitative agreement with the data (Fig. 3A, grey solid lines vs. coloured symbols). Briefly, both the deterministic approximations and data show that the motor rotation speed approaches an upper limit at low fields and motor rotation speed is zero at high fields. However, we do observe discrepancies in the intermediate regime: the data exhibit a more gradual change in speed than what was predicted by the deterministic approximations. This discrepancy may be attributed to thermal fluctuations. Indeed, near the stall point, taking account of thermal fluctuations causes a more gradual change in motor rotation speed (Supplement S4 and S5). However, even including thermal fluctuations into the model cannot fully account for the experimentally observed behaviour.

Another possible cause for the observed deviations between model and experimental data might be attributed to changes in motor torque during the experiment. To gain insight into how changes in motor torque would affect our observed bead rotation, we compute the deterministic motion of a magnetic bead at different motor torques ranging from 700–1400 pN·nm (Fig. 3A, from black to white). We observe that as the magnetic field increases, the experimental data intersects the higher motor-torque lines. This is indicative of an increase in motor torque with increased external load and is in accordance with results from previous studies that demonstrated an increase in the number of stators with increased load^29^,^30^. If the speed of stator recruitment is faster than the speed of magnet movement, the motor speed should indeed change more gradually with external load than for constant motor torque. In our experiments, however, we would expect stator exchange to result in fast (vertical) jumps in the motor speed, which we do not observe. Thus, we surmise that any changes with external load would have to occur more gradually and could have a different origin, such as by a change in local proton-motive force.

An alternative explanation for the observed deviations is based on the approximation for the magnetic torque (Supplement S2), which has only been shown to quantitatively predict data in the small angle approximation. Although the approximation has also been shown to qualitatively apply over full 2π rotation^21^, it has not been rigorously tested quantitatively. Therefore, we cannot exclude inaccuracies in the functional form of the model as a source of error in the fitted parameters. It is also possible that these deviations may simply result from considerable bead-to-bead variations, as previously observed^21^, or a tilted orientation of the anisotropy axis (Materials and Methods).

Direct fits (Supplement S3) to the data (Fig. 3, dashed lines) exhibit much better agreement with the data than the deterministic approximations. This can be understood from the number of free parameters used in fitting. In fitting, we fix *M*_*sat*_ and *B*_*0*_ and fit *τ*_*motor*_, *C* and *NV*, whereas in the deterministic approximations, we vary only *τ*_*motor*_ and fix all of the magnetic parameters, *M*_*sat*_, *B*_*0*_, *C*, and *NV*, to values found in the literature^21^,^24^. The fitted parameters give reasonable values for the motor torque (766 pN·nm in Fig. 3A and 988 pN·nm in Fig. 3B), but the fitted magnetic parameters deviate significantly from expected values (*NV* = 7.9·10^−3^ μm^3^, *C* = 0.35 kJ/m^3^ in Fig. 3A; *NV* = 7.8·10^−3^ μm^3^, *C* = 0.43 kJ/m^3^ in Fig. 3B; *NV* = (3.4 ± 3.1)·10^−2^ μm^3^, *C* = 0.33 ± 0.42 kJ/m^3^ (8 cells, including Fig. 3A and B) compared to *NV* = 2.4·10^−3^ μm^3^, *C* = 4.6 kJ/m^3^ in ref. 21).

Tracing the chronological order in which the data was taken (Fig. 3, colour coded from blue to red), we consistently observe that motors enter stall at the same field as they escape from stall, i.e., the blue-green data points approach zero speed at nearly the same field as the yellow-red data points start diverging from zero (an exception is discussed in Supplement S7). Interestingly, this observation indicates that there is no change in motor torque during stall. A previous study shows that the number of torque-generating stators varies with external load and that the BFM can recruit stators within a 300 s period of stall^29^. To try to resolve this apparent contradiction, we have to consider the time scale of stator recruitment. If stator recruitment occurs on a time scale that is faster than the time scale of load change (speed of magnet movement), such that the number of stators is equilibrated at each load, then our observations would be consistent with previously reported stator recruitment during stall or high load. When the external magnetic field increases, the load on the motor increases and the motor recruits stators. If the motor has already reached the maximum available number of stators by the time the external field stalls the motor, such that during the following period of stall no additional stators will join the motor complex, then the motor will escape from stall at the same field as it entered stall. In our experiments, the speed of magnet approach is ≈0.03 mm/s (or ≈30 s/mm), which means that it takes ≈200 s to go from almost free rotation to close to stall. Using the previously reported stator-exchange time^31^ of ≈30 s as a proxy for the stator-recruitment time scale, the time scale of magnet movement - and hence load change in our experiment - is slow compared to the stator recruitment time. As such, it appears plausible that the number of stators could have equilibrated at each load. In summary, our results suggest that either there is no additional stator recruitment during stall or that the stator recruitment occurs rapidly, on a time scale <200 s.

### Effects of forced motor rotation

Lastly, we investigate whether forced rotation by an external torque alters the properties of the flagellar motor by applying large torques and controlling the orientation of the magnetic field. Previous studies employing electrorotation and optical tweezers reported that motors often break after forced rotation in the backwards direction^15^,^19^,^32^. For these experiments, we used a non-switching strain that rotates exclusively in the counter clockwise direction.

**Figure 5:**
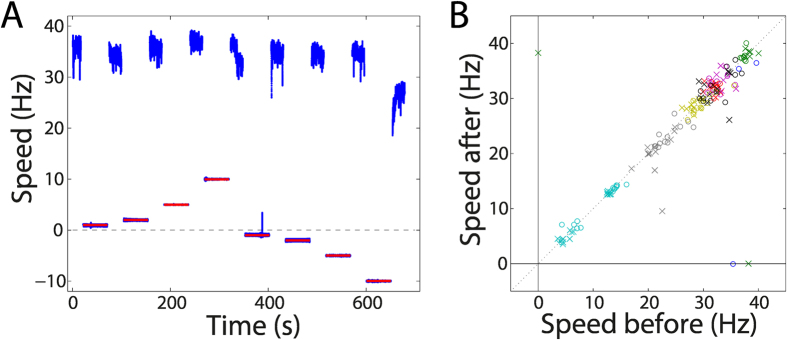
Recovery of motors after forced rotation. (**A**) Motor speed in time. The blue data points indicate the rotational speed of the bead averaged over a 0.2 s time window, and the red lines indicate the speed of the magnets. The speed of the bead between free and forced rotation is omitted. The speed of the magnets is only displayed during forced rotation of the bead, when the magnetic torque dominates. (**B**) Speed after forced rotation plotted against the speed before forced rotation for eight different motors. The measurement on the motor in (**A**) contributes eight data points in (**B**). Different colours correspond to different motors. Circles indicate forced rotation in the counter clockwise direction, the forward direction, and crosses indicate forced rotation in the clockwise direction, the backward direction.

To evaluate the effect of forced rotation on motor performance, we compared the drag-limited speed of the motor before and after forced rotation (Fig. 5). As an example, we considered a motor initially rotating at a drag-limited speed of approximately 35 Hz in a low magnetic field (1 mT; blue data, Fig. 5A). We then increased the external magnetic field to 340 mT, which is sufficient to completely stall the motor, and imposed a different rotation speed on the bead, and hence on the motor, by rotating the magnets (red data, Fig. 5A). After 50 s of forced rotation, we released the magnetic torque by decreasing the external field back to 1 mT and observed whether or not the motor is able to recover to its drag-limited speed (Fig. 5A). Subsequently, multiple cycles of forced rotation and release were performed. Negative enforced speeds indicate rotation in the clockwise direction, which is opposite to the (counter clockwise) rotation of the freely rotating motor. After all instances of forced rotation, the motor in the example trace shown was able to recover its initial speed of approximately 35 Hz (Fig. 5A), except after the last instance of forced rotation, when the motor only recovered up to approximately 25 Hz (Fig. 5A). Recovery to a lower speed than the initial speed could indicate loss of stators or other damage to the motor assembly.

We performed systematic measurements similar to the ones shown in Fig. 5A on a number of motors. Plotting the speed after forced rotation against the speed observed prior to forced rotation, we find that the large majority of points fall on or close to the diagonal. Thus, motors regularly recover their original drag-limited speed (Fig. 5B), with only minor variations in the speeds before and after forced rotation. Only a few points (<10%) fall below the diagonal, suggesting an incomplete recovery. In two of those cases (1.3% of all events) we observed no recovery of rotation after forced rotation (Fig. 5B, points on the *x*-axis). In one of those instances, we observed restoration of rotation ability through external rotation (Fig. 5B, point on the *y*-axis). These results suggest that under our experimental conditions, forced rotation in general does not damage or alter the flagellar motors, regardless of the speed and direction of the forced rotation (±0.1 to ±10 Hz for 20 or 50 s). The results contradict previous studies that report motors breaking after forced rotation^15^,^19^,^32^. The consistent recovery of the motors in our experiments could indicate the absence of destructive forces that were present in the previous techniques. By comparing the speeds before and after forced rotation for different cells (different colours in Fig. 5B), a cluster of points for each individual cell is observed. This suggests that the cells not only resume drag-limited rotation after forced rotation but that they retain memory of their state from before the forced rotation was imposed. The different speeds observed across the different cells possibly indicate a variation in the number of stators per motor, suggesting that stator number tends to remain conserved even during forced rotation. This forced-rotation experiment is another demonstration of the level of control that magnetic tweezers afford in this assay.

## Discussion

We have introduced magnetic tweezers as a powerful tool to study the rotational performance of bacterial flagellar motors. At low magnetic fields, our measurements are fully consistent with those obtained using tethered-bead assays. This demonstrates that our magnetic tweezers assay can be operated in a drag-based particle-tracking mode. Higher external fields (10–30 mT), which are easily achieved with the current permanent magnets, are sufficient to completely stall the motor, notably without inflicting photodamage or adverse heating. Measurements collected at stall allow us to quantify the stall torque, and the stall-torque values are in reasonable agreement with previously reported results and correspond with our low field measurements. In addition to the ability to adjust the external load, magnetic tweezers also provide us with the ability to impose the angular position upon the motor. In our experiments, forcing the motor to rotate forward and backward does not appear to adversely affect the motor. The ability to seamlessly adjust the external load on the motor allows us to switch between free rotation and stall and to probe the intermediate regime.

Over the entire range of magnetic fields, our experimental data are reasonably well described by a simple model. In the details, however, the model deviates from the data. Likely, this is due to known limitations in the construction of the model: for example, as the exact functional form of the magnetic potential is unknown, we approximate it by a sine function (Supplement S2). Use of this approximation could impact measurements of both the stall torque and the velocities as a function of magnetic field. Furthermore, the exact parameters of the magnetic beads inserted into the model are uncertain, in part because they have a high bead-to-bead variation, and in part because tilting of the anisotropy axis (Materials and Methods) may reduce their effective values. In principle, these magnetic parameters can be calibrated, as we have attempted by fitting the average speed versus field, but the results of this fit are again limited by the exactness of the model used to fit to. Possibly, an improved calibration approach would be to inactivate the motor following an experiment and to then measure the angular fluctuations. These fluctuations would then report on the angular stiffness of the system and allow for extraction of bead-specific magnetic parameters, accounting for the orientation of the anisotropy axis within an individual magnetic bead. After inactivation, the motor may be locked or declutched^17^. If the motor were locked, the stiffness of the system would equal the sum of the magnetic trap stiffness and the hook stiffness (Supplement S8), while if the motor were declutched, the rotor-shaft-hook-particle entity would be free to rotate and the stiffness of the system would equal that of the trap. We note that the more gradual change in motor speed versus magnetic field observed in the data relative to the model could alternatively be explained by a load-dependent change in the motor torque; however, if this were to occur, we would expect to measure a stall torque higher than the torque at drag-limited speed. If the load-dependent change was due to stator exchange, as previous studies have suggested, steps in the motor speed should occur. Overall, our investigation shows that extracting precise quantitative motor parameters, like torque, remains challenging even with magnetic tweezers; however, the power of this technique to noninvasively and actively apply torque on fast time scales holds independently of these considerations.

Our magnetic tweezers assay covers the full range of motor speeds from drag-limited speed to stall. For one-micron diameter beads, as used in our measurements, the speed ranges from 0–50 Hz. Use of smaller beads would allow a straightforward extension of this speed range because the rotational drag decreases with bead radius as 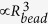. Smaller beads contain less magnetic material, however, and below a certain bead size the magnetic torque will likely no longer be sufficient to overcome the motor torque and stall the motor. However, taking into account that the rotational trap stiffness starts to saturate at fields above ≈150 mT^21^, a rough estimation suggests that beads with the same magnetic content per volume as the presently used MyOne beads could be at least five times smaller in volume than the MyOne beads.

The utility of magnetic tweezers in studying flagellar motors also depends on its mode of operation: angular-clamp mode (our assay) or torque-clamp mode. Magnetic tweezers naturally operate in angular-clamp mode, i.e., the magnetic beads tend to align with the external magnetic field, clamping the angular orientation of the beads. If the motor torque dominates over the magnetic torque, the bead sweeps through the magnetic potential and experiences an instantaneous magnetic torque that varies over the course of a revolution. The motor brakes due to a time-averaged magnetic torque over one revolution that opposes the motor. If the magnetic torque dominates over the motor torque, the angular clamp permits studies on changes in motor torque at stall and permits orienting the bead. Magnetic tweezers can also be operated in torque-clamp mode by requiring feedback to the field strength and orientation or by imposing fast field rotation causing asynchronous particle rotation^33^,^34^ or causing a phase lag between the field and the dipole inside the particle^35^. In a torque clamp, the magnetic torque is constant irrespective of the angular orientation of the bead. If the motor torque is larger than the magnetic torque, the magnetic torque brakes the motor. If the magnetic torque is larger than the motor torque, the magnetic torque forces the motor in reverse. Future experiments using the torque-clamp mode may complement the data described here and may reveal the mechanism of mechanosensing in BFMs and the BFM’s response to changes in torque and speed.

Our magnetic-tweezers assay equips us with a versatile tool to study rotary motors. This tool allows control over the strength and direction of the external magnetic field, and hence provides the user with the opportunity to bring the motor to a halt and even force motor rotation. The field strength can be varied from less than 1 mT to more than 300 mT in seconds. The orientation of the field can be changed on a time scale of 0.1 s. These features open the possibility of studying load-dependent motor dynamics on time scales not feasible before. Furthermore, magnetic tweezers are easy to combine with fluorescence techniques, broadening the scope of research on this dynamic motor complex even further.

## Materials and Methods

### Magnetic tweezers setup

A basic schematic of our magnetic tweezers configuration is depicted in Fig. 1. Briefly, we use a home-built inverted microscope with a 100× oil immersion objective (PLCN100XO, N.A. = 1.25, Olympus) positioned by a piezo-driven objective scanner (PIFOC P-726.1CD, Physik Instrumente). Two cubic magnets (W-05-N50-G, 5 × 5 × 5 mm^3^, Supermagnete) in vertical configuration^24^ generate the magnetic field. Motorized stages control both the height of the magnets above the sample plane (M-126.PD2, Physik Instrumente) and the orientation of the magnets (C-150.PD, Physik Instrumente) providing field-strength 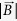 control on 1 s time scale and field-orientation 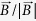 control on 0.1 s time scale. Flow cells are custom-made from two glass slides (24 × 60 mm^2^, Nr. 1, Menzel-Gläser) and a plastic paraffin film spacer (Parafilm M^®^, Bemis) sealing the sample channel. Liquids can be flushed in and out of the flow cells using a pump (11 Plus single syringe pump, Harvard Apparatus). Motorized actuators (8CMA06-25/15, Standa) are used to move the sample laterally. The sample plane is imaged using a high speed CMOS camera (MC1362, Mikrotron) and is illuminated using a red LED (LZ4-40R200, LED Engin). Data acquisition and device control are carried out using a LabVIEW program developed in-house (LabVIEW 2011, National Instruments)^36^,^37^.

### Magnet torque and force

In our magnetic tweezers setup, two permanent magnets generate the external magnetic field. As a consequence, the magnetic beads also experience, in addition to a magnetic torque, a magnetic force, which pulls the beads away from the surface towards the magnets. This force may for example tilt the magnetic “easy” axis (anisotropy axis) out of the horizontal plane (the plane of bead rotation) and hence reduce the effective dipole moment^38^. We note that the applied torque in the magnetic tweezers is related to the magnetic field^21^,^38^, whereas the force applied by the magnets is related to the gradient of the field^24^. The orientation of the anisotropy axis depends on the attachment point of the bead to the hook in the absence of any magnetic field, on the strength of the horizontally aligned field, i.e., on the torque on the bead, and on the gradient of this horizontally aligned field, i.e., on the force on the bead. In general, changing the distance of a fixed assembly of permanent magnets from the sample alters both the field and its gradient. The current magnet configuration, a 2 mm gap between vertically aligned magnets, was chosen to provide relatively high fields and low gradients. In combination with the MyOne beads used in our experiments, the force at stall, ≈30 mT, is well below 1 pN^24^,^39^. Nonetheless, the force could become more significant if higher fields are required, e.g. to be able to use and stall smaller beads. However, by altering the magnet configuration (e.g. increase the size of the gap between the magnets) or by using electromagnets^40^, it would be straightforward to maintain high fields while reducing the field gradients.

### *E. coli* strains and media

The switching strain used in our experiments is mTB47 (MTB32 + pET21a-BirA^41^). The following two non-switching strains were used: MTB22 (YS34^41^ +pDFB27^42^ + same flgE mutation as MTB32) and a derivative of MTB32^41^ obtained by a deletion of CheY (MTB32 + ΔCheY). The cells used here either have the stators under genomic expression (MTB47 and MTB32 + ΔCheY) or expressed from a plasmid (MTB22), in which case the inducer, 5 mM arabinose, was only present during growth but absent during measurements. Cells of all strains are grown in tryptone broth (10 g/l tryptone, 5 g/l sodium chloride) at 30 °C with shaking at 250 RPM for 5.5 hours to an OD_600_ (optical density at 600 nm) of approximately 0.6. Cells are washed by centrifuging 1 ml of cell culture at 10,000 rpm for 1 min and resuspending in 1 ml of motility buffer (10 mM sodium phosphate buffer, 85 mM sodium chloride, 0.1 mM EDTA, pH 7.0).

### Experimental procedure

A flow cell is mounted on the stage. The sample is prepared in the absence of the permanent magnets. First, the flow cell is filled with poly-L-lysine (PLL, Sigma-Aldrich, P4707) to coat the bottom glass slide of the flow cell and enable *E. coli* cell adherence. After 15 minutes, the PLL is flushed out and a solution of *E. coli* cells in motility buffer is added. The flow cell is incubated for 15 minutes after which non-attached *E. coli* cells are flushed out. Streptavidin-coated superparamagnetic beads (Dynabeads, MyOne, 1 μm diameter) are attached to the biotinylated hooks of the fixed bacterial cells by incubation for 15 minutes. Non-attached beads are removed from the flow cell by flushing. All experiments are performed in motility buffer. The permanent magnets are mounted above the flow cell before measurements commence.

### Magnetic field strength

The magnetic field strength depends on the magnet configuration^24^ and the distance between magnets and sample^24^. The conversion from magnet-sample distance to magnetic field strength is performed using a look-up table. The look-up table was generated by computation of the Biot-Savart law, using the experimentally determined value for the remanent magnetization of the magnets^24^. The computed fields were shown to be in excellent agreement with direct Hall probe measurements^24^.

### Image analysis

Images of beads attached to active motors are recorded using video microscopy at 500 Hz and a tracking algorithm is used to determine the bead position in each frame in real time^37^,^43^. Next, the (*x, y*)-positions of the bead are plotted revealing the bead’s trajectory. In the absence of an external magnetic field, the beads are assumed to trace out circular trajectories. Ideally, the motor is located on top of the cell, i.e., the rotation axis of the motor is parallel to the *z*-axis and the bead rotates in the (*x, y*)-plane. If the rotation axis of the motor is not parallel to the *z*-axis, however, the bead trajectory contains a *z*-component and the bead will appear to trace out an ellipse in the (*x, y*)-plane, the ellipse being the corresponding projection of a circle. Trajectories with no or only little motion in *z* appear circular in the (*x, y*)-plane and have (close to) zero eccentricity. Trajectories with a more significant *z*-components are marked by larger values for the eccentricity of the ellipse in the (*x, y*)-plane. Motors with beads attached that trace out ellipses with a major-to-minor-axis ratio close to unity (eccentricity less than 0.75) are selected, and the data are algebraically fitted to an ellipse using a custom routine in MATLAB (adapted from MATLAB Central, File ID: #3215). Each experimentally determined (*x, y*)-position is projected to its nearest point on the fitted ellipse to convert the (*x, y*)-position to rotation angle.

## Additional Information

**How to cite this article**: van Oene, M. M. *et al*. Applying torque to the *Escherichia coli* flagellar motor using magnetic tweezers. *Sci. Rep.* **7**, 43285; doi: 10.1038/srep43285 (2017).

**Publisher's note:** Springer Nature remains neutral with regard to jurisdictional claims in published maps and institutional affiliations.

## Supplementary Material

Supplementary Information
