# Corrigendum: Applying torque to the *Escherichia coli* flagellar motor using magnetic tweezers

**DOI:** 10.1038/srep46980

**Published:** 2018-05-18

**Authors:** Maarten M. van Oene, Laura E. Dickinson, Bronwen Cross, Francesco Pedaci, Jan Lipfert, Nynke H. Dekker

Scientific Reports 7: Article number: 4328510.1038/srep43285; published online: 03 07 2017; updated: 05 18 2018

The Acknowledgements section in this Article is incomplete.

“We thank Seungkyu Ha, Yera Ye. Ussembayev, Richard Janissen, and Hubertus J. E. Beaumont for discussions, Richard M. Berry and Ren Lim for providing the strains, and Theo van Laar for the remaining help with the bacteria. This work is supported by NanoNextNL, a micro and nanotechnology consortium of the Government of the Netherlands and 130 partners, and by the Foundation for Fundamental Research on Matter (FOM).”

should read:

“We thank Seungkyu Ha, Yera Ye. Ussembayev, Richard Janissen, and Hubertus J. E. Beaumont for discussions, Richard M. Berry and Ren Lim for providing the strains, and Theo van Laar for the remaining help with the bacteria. FP was supported by the European Research Council under the European Union’s Seventh Framework Program (FP/2007–2013)/ERC Grant 306475. This work is supported by NanoNextNL, a micro and nanotechnology consortium of the Government of the Netherlands and 130 partners, and by the Foundation for Fundamental Research on Matter (FOM).”

