# Supplementary material for: Applying torque to the Escherichia coli flagellar motor using magnetic tweezers
Source: Sci Rep. 2017 Mar 7;7:43285. doi: 10.1038/srep43285 (PMC5339722; doi:10.1038/srep43285)
Supplement: Supplementary Information [file srep43285-s1.pdf]

Supplementary Information for:

Applying torque to the *Escherichia coli* flagellar motor using magnetic tweezers

Maarten M. van Oene[1], Laura E. Dickinson[1], Bronwen Cross[1], Francesco Pedaci[1,2], Jan Lipfert[3], and Nynke H. Dekker[1,*]

[1]Department of Bionanoscience, Kavli Institute of Nanoscience, Delft University of Technology, Lorentzweg 1, 2628 CJ Delft, Netherlands

[2]Present address: Department of Single-Molecule Biophysics, Centre de Biochimie Structurale, UMR 5048 CNRS, Montpellier, France

[3]Department of Physics, Nanosystems Initiative Munich, and Center for NanoScience, LMU Munich, Amalienstrasse 54, 80799 Munich, Germany

S1 MOTOR-DRIVEN ROTATION OF A MAGNETIC BEAD IN A MAGNETIC POTENTIAL 2

S2 ANALYTICAL APPROXIMATION FOR THE MAGNETIC TORQUE 2

S3 AVERAGE SPEED IN THE DETERMINISTIC APPROXIMATION 3

S4 AVERAGE SPEED IN THE STOCHASTIC DESCRIPTION 4

S5 NUMERICAL SIMULATION OF ANGULAR TRACES 5

S6 EXPERIMENTAL TRACES OF THE BEAD POSITION 10

S7 MOTOR LOSING FUNCTION 12

S8 DOUBLE SPRING SYSTEM: MOTOR-BEAD-MAGNETIC TRAP 13

S9 INSTANTANEOUS SPEED IN THE DETERMINISTIC APPROXIMATION 14

SUPPLEMENTARY REFERENCES 15

S1 Motor-driven rotation of a magnetic bead in a magnetic potential

The equation of motion for a magnetic bead spun around by a rotary motor in an external magnetic field in the overdamped limit, relevant in our case, is given by:

$$0 = \tau_{magnet} + \tau_{motor} + \tau_{drag} + \tau_{thermal}, \qquad \textbf{Equation S1}$$

where τ_{magnet} is the torque due to the interaction between the magnetic field of the magnets and the dipole of the bead; τ_{motor} is the motor torque and quantity of interest; $\tau_{drag} = -\gamma_{bead}\omega_{bead}$ is the drag torque on the bead, where γ_{bead} is the rotational drag coefficient in bulk, and ω_{bead} is the angular speed; and $\tau_{thermal} = \sqrt{2k_B T\gamma_{bead}}\eta(t)$ is the torque due to thermal fluctuations on the bead, where $\eta(t)$ is a white-noise process with properties [1]:

$$\langle\eta(t)\rangle = 0;\ \langle\eta(t)\eta(t')\rangle = \delta(t-t'), \qquad \textbf{Equation S2}$$

where δ is Dirac's delta function. We perform numerical simulations and analytical calculations of the simple model in **Equation S1** for a quantitative description of our assay. In the modelling, we assume for simplicity that the motor torque is independent of its rotational speed. This assumption is motivated by the fact that the motor torque is reported to be roughly constant in the plateau of its torque-speed curve (in *E.coli*, the motor torque decreases by ≈ 10% between 0 and 175 Hz [2]). A change in motor torque (e.g. due to stator exchange or other external factors) would appear as a jump between curves simulated at different (constant) motor torques, and can be accounted for by these jumps.

S2 Analytical approximation for the magnetic torque

As the motor torque is the quantity of interest, the only term in **Equation S1** not discussed yet is the magnetic torque. A previous report [3] has shown that the magnetic potential for a MyOne bead in magnetic tweezers is π periodic. An obvious and simple choice is then to assume $V(x) \propto -\cos(2x)$, where $x = \theta - \theta_0$, because $V(x)$ is π periodic and has a minimum at $x = 0$. The analytical calculations (**Supplement S3** and **S4**) approximate the magnetic torque accordingly as:

$$\tau_{magnet} = -\tau_0 \sin\big(2(\theta - \theta_0)\big), \qquad \textbf{Equation S3}$$

where τ_0 is the maximum magnetic torque, θ is the angular orientation of the anisotropy axis inside the magnetic bead, and θ_0 is the orientation of the external magnetic field. In such a potential, the trap stiffness near the bead's equilibrium position θ_0 is $\kappa_{trap} = -\partial\tau_{magnet}/\partial\theta = 2\tau_0$, from which it follows that the maximum magnet torque is $\tau_0 = \kappa_{trap}/2$. From previous reports [3], we know that near the bead's equilibrium position, $\kappa_{trap} = NV\ C\ B\ M/(C + B\ M)$, where NV is the effective volume of superparamagnetic nanoparticles inside the bead, B is the magnetic field, and $M(B)$ is the magnetization, which depends on the field B. We make three remarks about this sine approximation for the magnetic torque: first, the potential for aligned (an approximation itself) superparamagnetic nanoparticles looks more like a skewed sine and approaches an actual sine function only in the low and high field limits; second, although the magnetic torque averaged over one revolution is zero ($\int_0^{2\pi} A\sin(2x)dx = 0$ with $A = -\tau_0$ and $x = \theta - \theta_0$), the relevant parameter here is the time-averaged magnetic torque over one revolution

($\int_{t(x=0)}^{t(x=2\pi)} A\sin\big(2x(t)\big)dt$), which is nonzero for nonzero fields; third, the magnetic torque reduces the time-averaged motor speed, but the instantaneous motor speed at certain bead orientations actually increases compared to the drag-limited speed (**Supplement S9**), because at those orientations the motor torque and magnetic torque work in the same direction. In addition to this sinusoidal approximation, the analytical calculations assume deterministic behaviour, i.e., $\tau_{thermal} = 0$. This deterministic approximation (**Supplement S3**) converges to the stochastic solution (**Supplement S4**) in the low and high field limits. The effect of noise is most apparent near the critical point, stall, where motor torque and magnetic torque are similar in magnitude.

S3 Average speed in the deterministic approximation

In the deterministic approximation, the effect of thermal fluctuations is ignored, i.e., $\tau_{thermal} = 0$. The noise-free equation of motion for a magnetic bead spun around by a rotary motor in an external magnetic field in the overdamped limit is given by:

$$-\tau_{drag} = \tau_{magnet} + \tau_{motor}, \qquad \textbf{Equation S4}$$

where each of the terms is as indicated in the main text. We substitute the appropriate parameters:

$$\gamma_{bead}\omega_{bead} = -\tau_0 \sin\big(2(\theta - \theta_0)\big) + \tau_{motor}, \qquad \textbf{Equation S5}$$

where $\omega_{bead} = \dot{\theta}$. When the orientation of the external magnetic field is fixed, as in our current experiments, $\frac{d}{dt}(\theta - \theta_0) = \omega_{bead}$, and we rewrite the noise-free equation of motion as:

$$\dot{x} = -C_1 \sin(2x) + C_2. \qquad \textbf{Equation S6}$$

where $x = \theta - \theta_0$, $C_1 = \tau_0/\gamma_{bead}$, and $C_2 = \tau_{motor}/\gamma_{bead}$.

For $C_2 > C_1$, the torque signal is a periodic function with period [4]:

$$T = \frac{\pi}{\sqrt{C_2^2 - C_1^2}}. \qquad \textbf{Equation S7}$$

The speed of rotation in Hertz is then given by:

$$f_{rot} = \frac{1}{2T} = \frac{\sqrt{C_2^2 - C_1^2}}{2\pi}, \qquad \textbf{Equation S8}$$

and the corresponding angular speed in rad/s is:

$$\dot{x} = 2\pi f_{rot} = \sqrt{C_2^2 - C_1^2}. \qquad \textbf{Equation S9}$$

When the magnetic torque is negligible, i.e, $C_1 \ll C_2$, the angular speed is simply $\dot{x} = \sqrt{C_2^2} = \tau_{motor}/\gamma_{bead}$. As the magnetic torque increases, the motor brakes, and once the magnetic torque is equal in magnitude to the motor torque, $C_1 = C_2$, the motor stalls, so $\dot{x} = \sqrt{C_2^2 - C_1^2} = 0$. This expression for the angular speed (**Equation S9**) provides a convenient analytical approximation for the speed-versus-field curve, because $\dot{x}\left(C_1\big(\tau_0(B)\big)\right)$.

For fitting the average speed versus magnetic field, we employ **Equation S9**, where $C_2 = \tau_{motor}/\gamma_{bead}$ and $C_1 = \tau_0/\gamma_{bead}$ with $\tau_0 = \kappa_{trap}/2$, $\kappa_{trap} = NV\,C\,B\,M/(C + B\,M)$, and $M(B) = M_{sat}\left(\coth\left(\frac{B}{B_0}\right) - \frac{B_0}{B}\right)$. We calculate γ_{bead}, and set M_{sat} = 43.3 kA/m and B_0 = 12 mT [5]; the fitting parameters are NV, C, and τ_{motor}.

S4 Average speed in the stochastic description

We have an analytical expression for the deterministic approximation (**Equation S9**), but we would also like to understand the effect of thermal fluctuations. We consider the equation of motion (**Equation S6**), which describes a tilted periodic potential. The periodic potential is:

$$V(x) = -\frac{1}{2}\tau_0 \cos(2x), \qquad \textbf{Equation S10}$$

where $x = \theta - \theta_0$. An external force F tilts this periodic potential; in our system the tilting force F is the motor torque. The tilted periodic potential $U(x)$ is:

$$U(x) = V(x) - Fx = -\frac{1}{2}\tau_0 \cos(2x) - \tau_{motor}x. \qquad \textbf{Equation S11}$$

Without the assumption of a noise-free system as in **Supplement S3**, Stratonovich showed that the time-averaged speed is described by [6]:

$$\langle \dot{x} \rangle = \frac{D_0\left[1 - \exp\left(-\frac{Fl}{k_BT}\right)\right]}{l\,\langle I_\pm \rangle}, \qquad \textbf{Equation S12}$$

where $D_0 = k_BT/\gamma_{bead}$, $l = \pi$, and we arbitrarily choose to use $\langle I_- \rangle$, which is given by [6]:

$$\langle I_- \rangle = \frac{1}{\pi}\int_0^\pi \left[\frac{1}{\pi}\int_0^\pi \exp\left(-\frac{U(x) - U(x+y)}{k_BT}\right) dy\right] dx. \qquad \textbf{Equation S13}$$

To understand the effect of thermal fluctuations, we compare the deterministic solution (**Equation S9**) to the stochastic solution (**Equation S12**) at different temperatures, where we perform the integration in **Equation S13** numerically (**Figure S1**). All curves overlap in the low and high field limits, as they should. For decreasing temperatures, the curves move towards the analytical solution of **Equation S9**, as the thermal fluctuations reduce to the curve where they are not considered (**Equation S9**). The stochastic approximation (**Equation S12**) includes thermal effects, however, it is less convenient to use than the deterministic approximation (**Equation S9**) because of the integral $\langle I_\pm \rangle$ (**Equation S13**). Both the deterministic and stochastic approximations still assume a sinusoidal description of the magnetic potential (**Equation S10**), which might be incorrect. Therefore we also perform numerical simulations (**Supplement S5**).

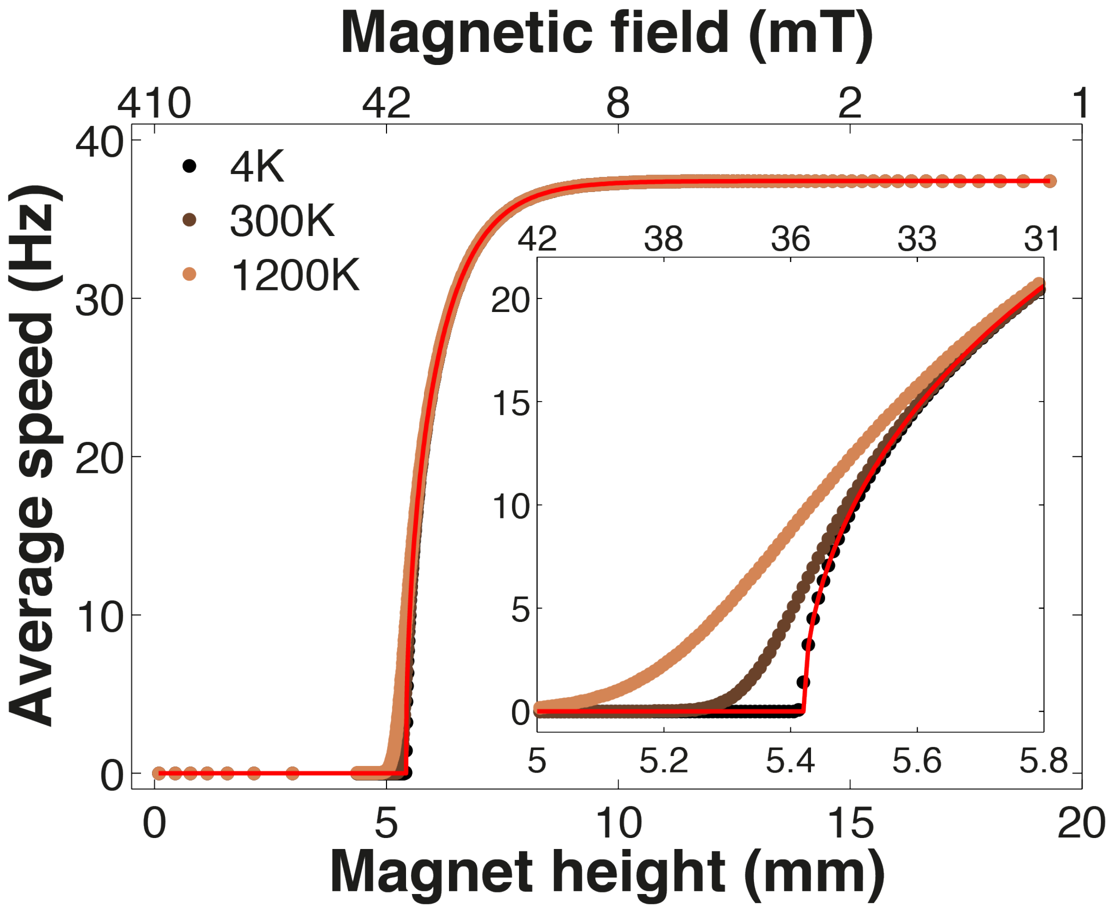

Figure S1 Three stochastic and one deterministic solution for the average motor speed at different magnet distances to the sample (vertical magnet configuration with 2 mm gap size between magnets). The data points display the results of the stochastic solution (**Equation S12**) at the designated temperatures: 4, 300, and 1200 K. The red line is the result of the deterministic solution (**Equation S9**). The inset is a zoom-in near $C_1 = C_2$, where the stochastic solutions deviate most from the deterministic solution.

S5 Numerical simulation of angular traces

We numerically simulate angular traces of a magnetic bead spun around by a rotary motor in an external magnetic field. We repeat the simulations at different magnetic field strengths, and extract the average speed, the mean angle, and the standard deviation in the angle. Starting with the equation of motion, filling in all terms, and rewriting, we obtain:

$$\theta(t+\Delta t) = \theta(t) + \left(\frac{\tau_{magnet} + \tau_{motor} + \tau_{thermal}}{\gamma_{bead}}\right)\Delta t, \qquad \textbf{Equation S14}$$

where τ_{magnet} is the magnetic torque, explained below, τ_{motor} is the motor torque, and $\tau_{thermal} = r_n\sqrt{2k_B T \gamma_{bead}/\Delta t}$, where r_n is a random number drawn from a normal distribution with zero mean and unit standard deviation.

The magnetic torque τ_{magnet} is calculated as described previously [3]. Summarizing, we calculate the torque on a single superparamagnetic nanoparticle, which depends on the external magnetic field and the angle θ_{NP} between anisotropy axis and field. To obtain the torque on a single bead, we assume the bead consists of a polymer matrix

with embedded, identical, superparamagnetic nanoparticles that are all aligned in the same direction, so $\tau_{magnet} = N_{NP}\tau_{NP}$. We extract the torque on the bead as a function of field and orientation θ_{NP} from a lookup table during the simulations to reduce computation time.

Two examples of simulated angular traces are depicted in **Figure S2** and **Figure S3**. We simulate the angle θ, and we deduce the corresponding x and y positions using the parametric equations for an ellipse, arbitrarily selecting a length for the major and minor axes. Two examples are shown: one at 1 mT (**Figure S2**), where the speed is essentially only limited by drag; and one at 24 mT (**Figure S3**), when the motor nearly stalls. We display the x and y positions first to facilitate comparison with the experimental datasets (**Figure S7** and **Figure S8**).

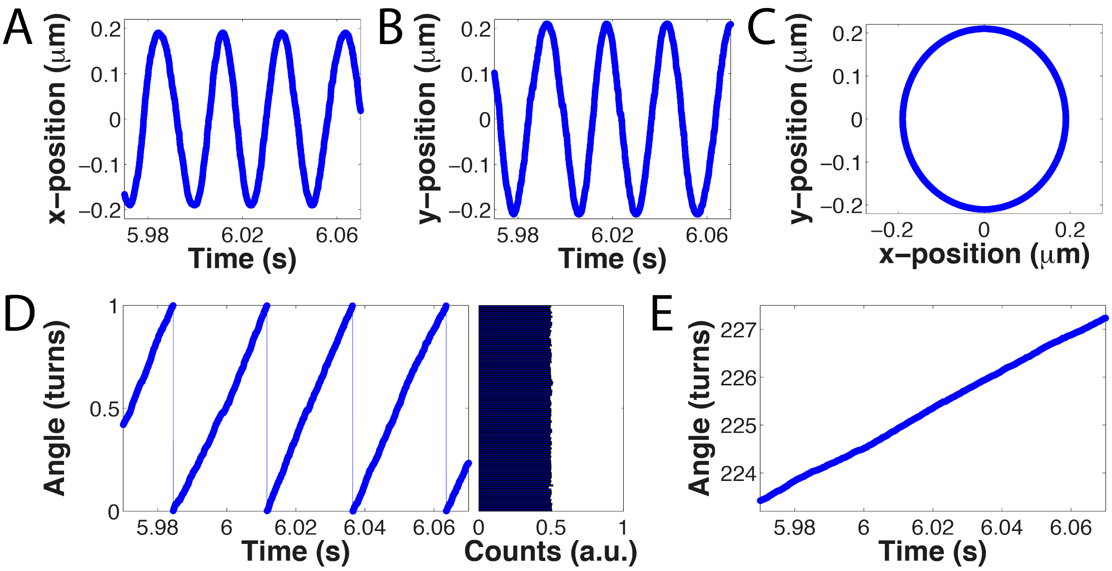

Figure S2 Numerically simulated trace of a magnetic bead spun around by a rotary motor in an external magnetic field at 1 mT. The full trace is 25 s long. Shown are the x position vs. time (A), y position vs. time (B), the (x, y) positions over the full trace (C), the “wrapped” angle vs. time and its full-trace histogram (D), and the “unwrapped” angle vs. time (E).

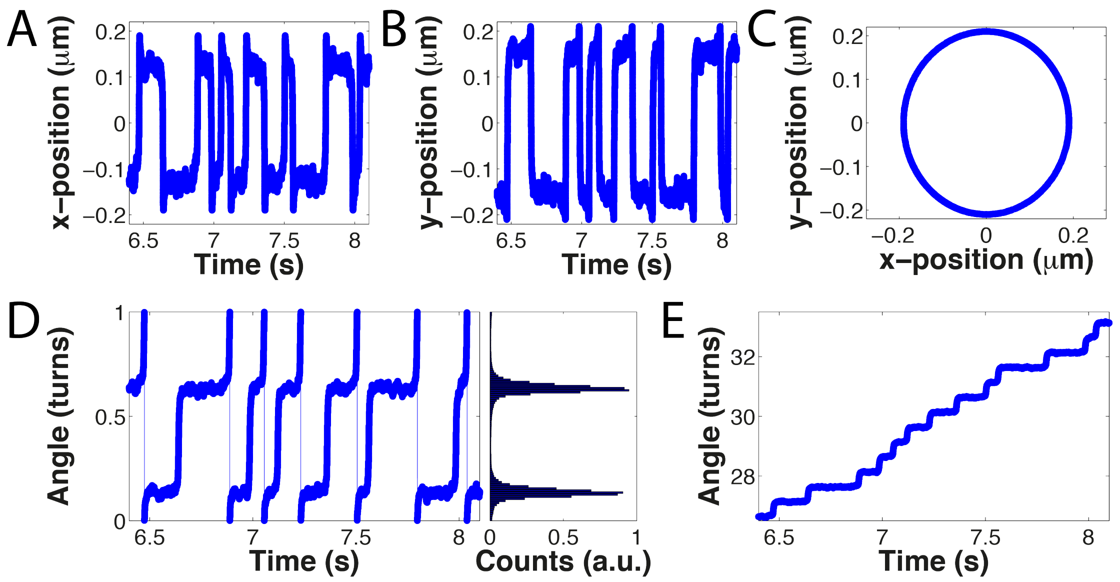

Figure S3 Numerically simulated trace of a magnetic bead spun around by a rotary motor in an external magnetic field at 24 mT. The full trace is 25 s. Shown are the x position vs. time (A), y position vs. time (B), the (x, y) positions over the full trace (C), the "wrapped" angle vs. time and its full-trace histogram (D), and the "unwrapped" angle vs. time (E).

Traces simulated over a wide range of fields are used to extract the average motor speed, the bead orientation during stall, and the angular fluctuations of the bead during stall versus magnet distance. These extracted quantities are plotted in **Figure S4**, **Figure S5**, and **Figure S6**, respectively. These figures serve as comparison to **Figures 3** and **4** of the main text. We fit the simulations with the analytical approximations: the average speed of the rotating bead in **Figure S4** with **Equation S9** and the mean angle in **Figure S5** with **Equation 5** of the main text.

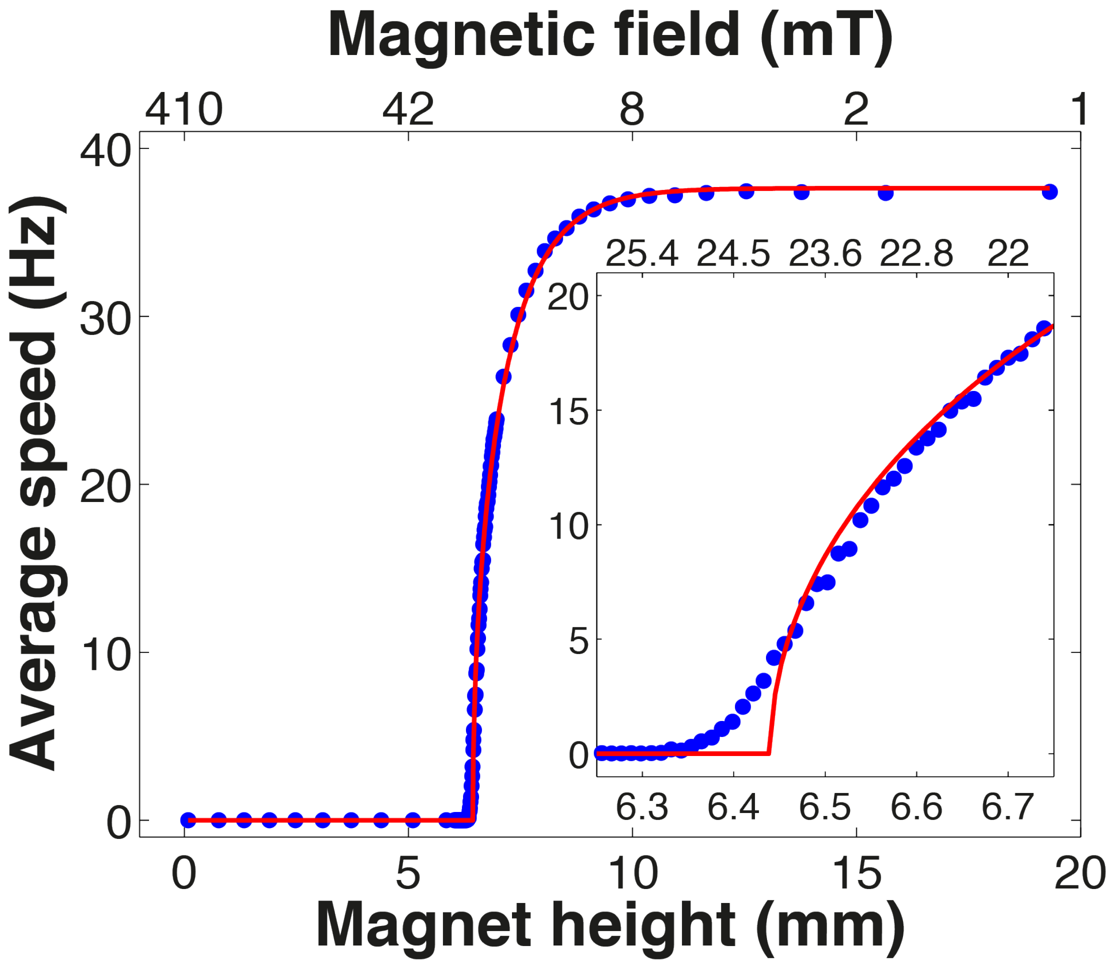

Figure S4 Numerical simulation of the average motor speed versus magnet height. The blue data points are the numerically simulated data, and the red line is the fit to the analytical approximation (**Equation S9**). The fit parameters are $NV = 4.8 \cdot 10^{-3}$ μm^3, $C = 1.7$ kJ/m^3, and $\tau_{motor} = 1.0 \cdot 10^3$ pN·nm. The inset shows a zoom-in near the stall field, where the effect of thermal fluctuations is apparent.

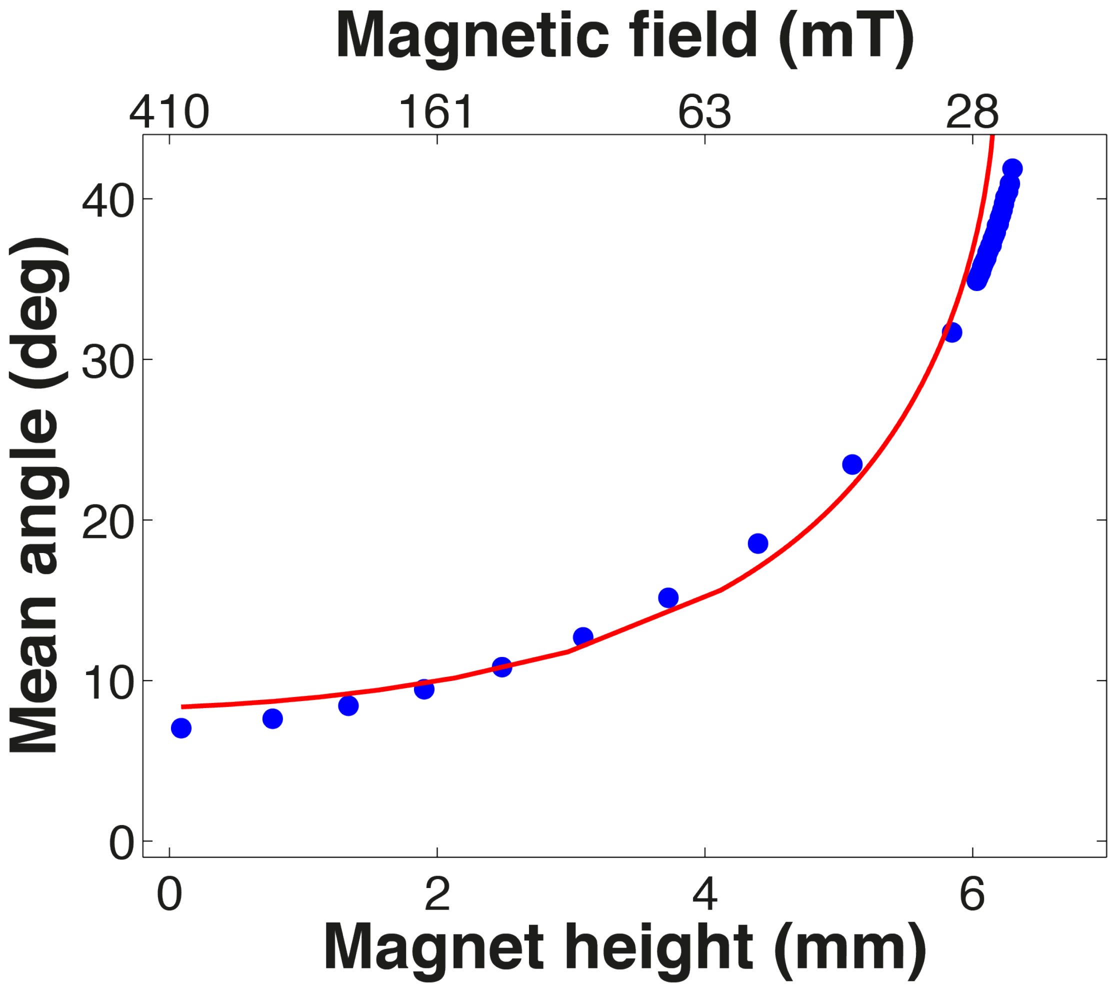

Figure S5 Numerical simulation of the mean bead orientation versus magnet height. The blue data points are the numerically simulated data, and the red line is the fit to the analytical approximation (**Equation 3** of the main text). The fit parameters are $\theta_0 = 3.8°$, and $\tau_{motor} = 691$ pN·nm.

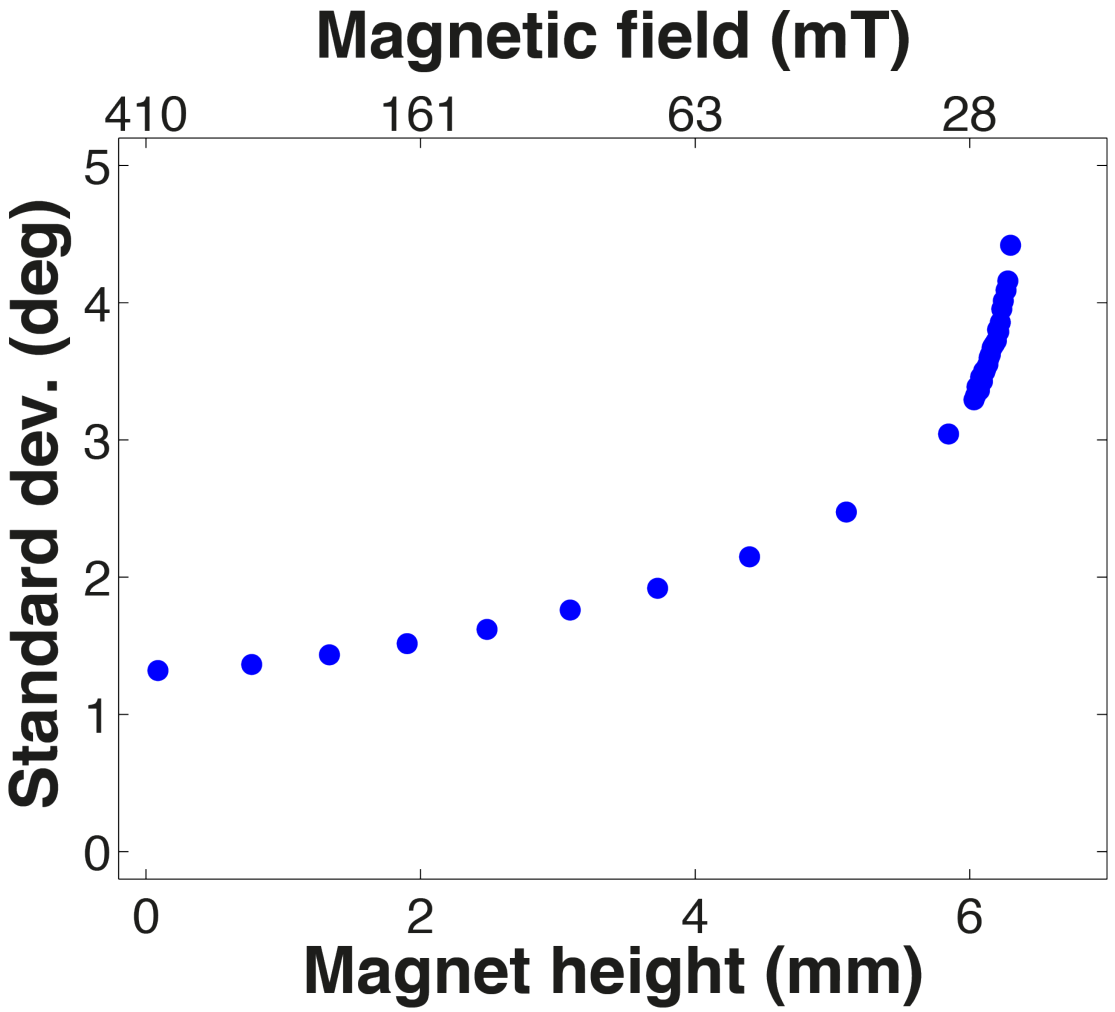

Figure S6 Numerical simulation of the standard deviation in bead orientation versus magnet height. The blue data points are the numerically simulated data.

In **Figure S5** and **Figure S6**, we observe that the mean and standard deviation in the angle do not go to zero for high fields, but level off at a nonzero value. This is because the trap stiffness saturates at high fields. The expected mean angle at high fields is $\frac{1}{2}\text{asin}\left(\tau_{motor}/\tau_{0,max}\right) \approx 5°$, and the expected standard deviation at high field is $\approx 1°$.

From **Figure S4** and **Figure S5**, we observe that the analytical expressions fit the numerical simulations reasonably well. This good agreement makes it possible for us to employ the analytical expressions to compare to the experimental data instead of the numerical simulations. As a concomitant advantage, the analytical expressions are more convenient to use than the numerical simulations, because the calculations are faster.

S6 Experimental traces of the bead position

In our magnetic tweezers assay, a tracking algorithm determines the position of the bead in each of the recorded video images. We convert the extracted x and y positions to an angular position using a fit to the mathematical function that describes an

ellipse. Two examples of such angular traces are depicted in **Figure S7** and **Figure S8**: one at 1 mT when the speed is essentially only limited by drag (**Figure S7**) and one at 9 mT when the motor nearly stalls (**Figure S8**).

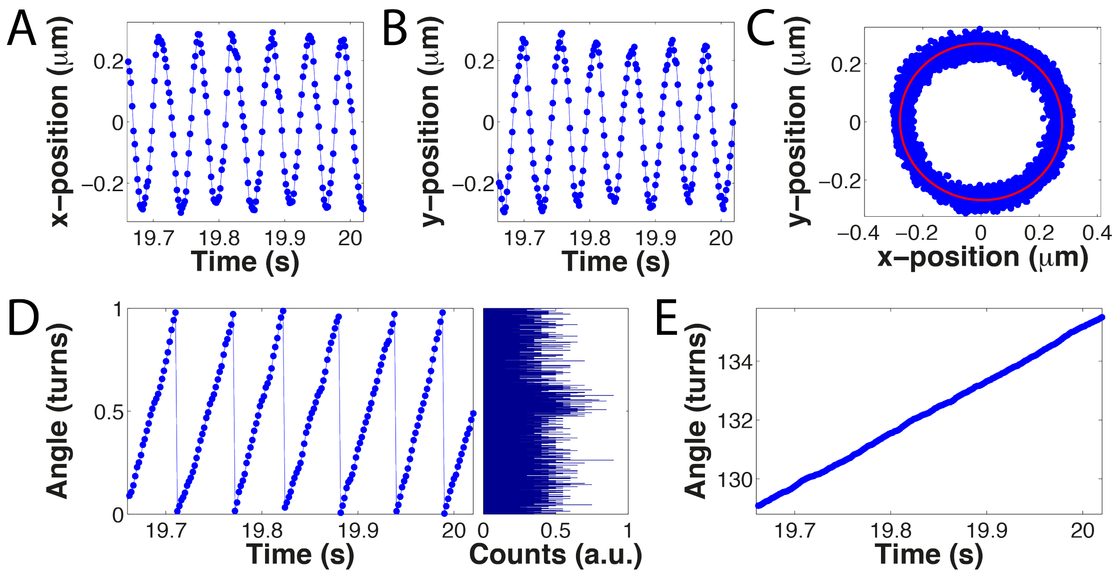

Figure S7 Experimentally recorded trace of a magnetic bead spun around by the bacterial flagellar motor in an external magnetic field at 1 mT. The full trace is approximately 15 s. Shown are the x position vs. time (A), y position vs. time (B), the (x, y) positions over the full trace with fitted ellipse (C), the "wrapped" angle vs. time and its full-trace histogram (D), and the "unwrapped" angle vs. time (E).

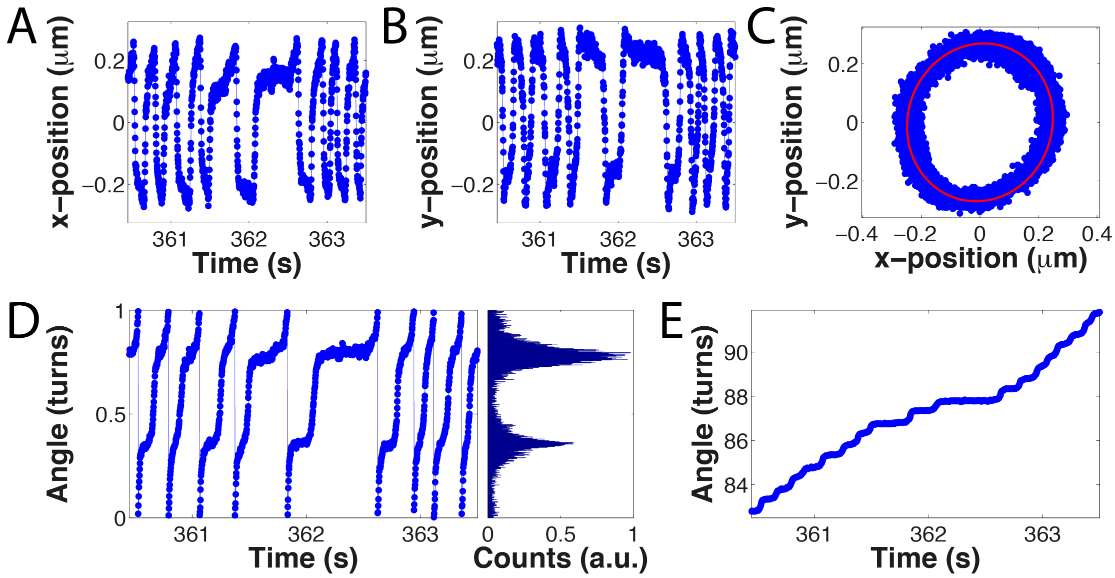

Figure S8 Experimentally recorded trace of a magnetic bead spun around by the bacterial flagellar motor in an external magnetic field at 9 mT. The full trace is approximately 30 s. Shown are the x position vs. time (A), y position vs. time (B), the (x, y) positions over the full trace with fitted ellipse (C), the "wrapped" angle vs. time and its full-trace histogram (D), and the "unwrapped" angle vs. time (E).

S7 Motor losing function

In a particular measurement, we found the motor to lose some and later on most of its torque-generating power (**Figure S9**). We find such a case to be exceptional.

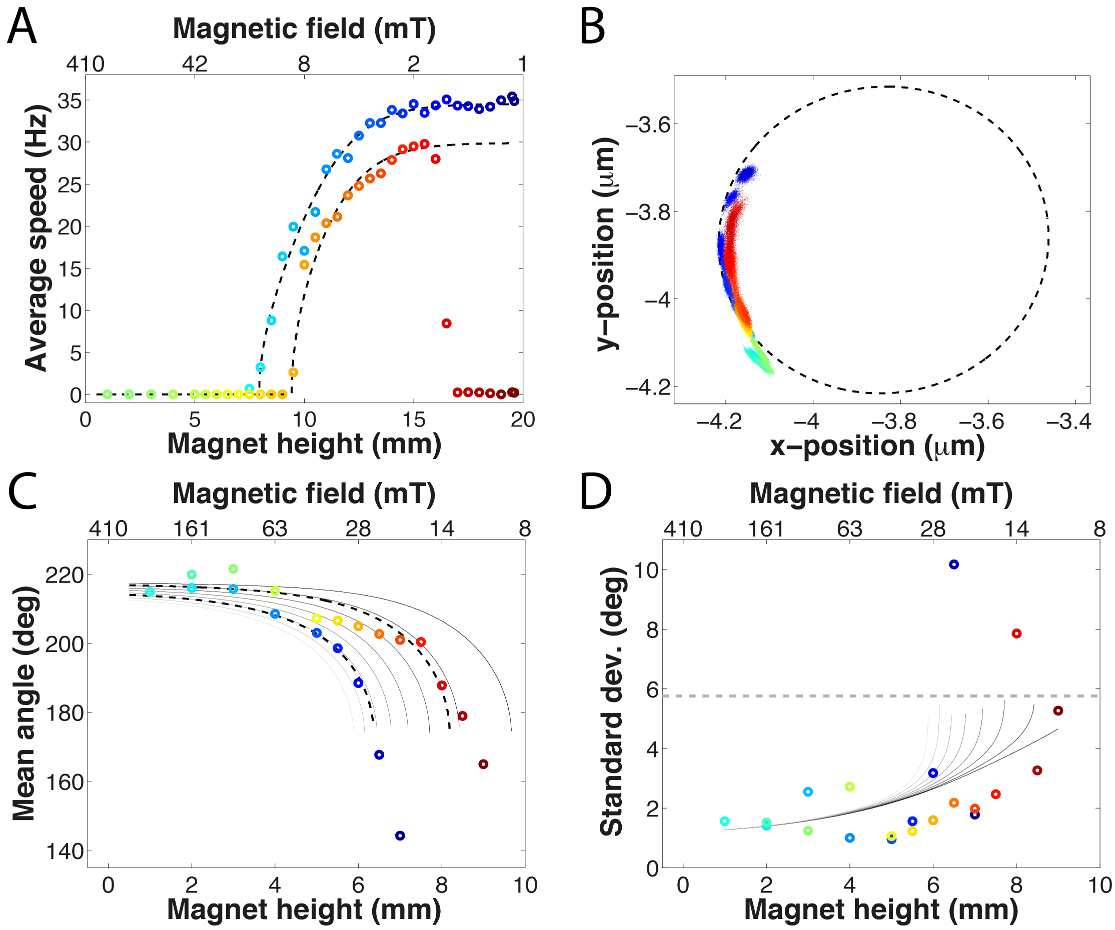

Figure S9 Motor losing function during measurement. During the experiment, the magnet distance to the sample is first decreased in discrete steps and then increased again in the same fashion, as indicated by the color coding going from blue to red. In (A), the color coding starts at a magnet height of 19.6 mm, then goes down to 1 mm and back up to 19.6 mm. In (B)-(D), only part of the data in (A) is used: the color coding starts at a magnet height of 7 mm, then goes down to 1 mm and back up to 9 mm. (A) Average motor speed versus magnet height. The dashed black lines are fits to the analytical approximation (**Equation S9**). The fit parameters are $NV = 7.0 \cdot 10^{-2}$ μm^3, $C = 30$ J/m^3, and $\tau_{motor} = 923$ pN·nm for the upper curve and $\tau_{motor} = 799$ pN·nm for the lower curve. (B) Bead position during stall. The dashed line is an ellipse fit to rotation data of the same motor. (C) Mean angular position at different magnet heights. The grey-colored lines are coplotted simulations (**Equation 3** of the main text), only different in the motor torque. The motor torque increases from black to white from $\tau_{motor} = 100$ pN·nm to 800 pN·nm with $\theta_0 = 218°$. The black dashed lines are fits to the data with fitted parameters $\theta_0 = 218°$ and $\tau_{motor} = 229$ pN·nm for the upper curve and $\tau_{motor} = 626$ pN·nm for the lower curve. (D) Standard deviation in angular position at different magnet heights. The grey-colored lines are coplots of **Equation 4** of the main text with $\kappa_{hook} = 400$ pN·nm/rad [7,8], κ_{trap} is based on previous results [3], and τ_{motor} as in (C). The dotted line indicates the fluctuations under a minimal stiffness, only due to the hook for $\kappa_{hook} = 400$ pN·nm/rad.

In this case, the motor torque reduces during stall. This reduction can be seen from **Figure S9**(A) and (C). At the start of the experiment (blue data in the average speed-versus-magnet height plot), the motor rotates at approximately 35 Hz. As the magnetic field in the sample plane increases, the average speed decreases until the motor stalls. During stall, the magnetic field strength does not affect the average

speed; the speed remains 0 Hz. When reducing the magnetic field strength again, the motor only escapes from stall at lower field strength than it entered stall, indicating a reduced motor torque. Further, after escaping from stall, the motor does not recover to its initial speed, again indicating a reduced motor torque. **Figure S9**(C) shows that the motor enters stall (blue data) at a higher field than it escape from stall (red data). In addition, it requires higher field strengths to force the bead to align with the field than when the magnetic torque is reduced again, indicating a higher motor torque during the first part of the period of stall than during the second part.

When the magnetic field becomes less than 2 mT, the motor speed suddenly drops to less than 0.5 Hz. As if the reduced motor torque after stall was an indicator for some sort damage to the motor, the motor speed reduces just when the magnetic torque starts to become negligible (≈ 1-2 mT). The change in speed happens instantaneously on the time scale of our measurement; the average speed at magnet height 16.5 mm is 8.4 Hz, because the sudden change in speed happened during that measurement point, and we averaged over the full measurement point.

S8 Double spring system: motor-bead-magnetic trap

In our experimental system (**Figure 1** of the main text), the thermal fluctuations of the magnetic bead are constrained by the magnetic trap and by the flagellar system of motor and hook. This system is described as a double spring system with two torsional springs, the magnetic trap and the hook, working in parallel (**Figure S10**). Because the two springs work in parallel, the stiffness of the system is simply the sum of the two spring constants, i.e., $\kappa_{system} = \kappa_{trap} + \kappa_{hook}$. The amplitude of the angular fluctuations depends on the thermal energy $k_B T$ and on the stiffness of the system κ_{system}, and is described by equipartition theorem, i.e., $\langle \theta^2 \rangle = k_B T / \kappa_{system}$.

In our magnetic tweezers setup, κ_{trap} depends on the external magnetic field, and can be varied from $\approx 0 - 10\ pN \cdot \mu m/rad$. The hook stiffness κ_{hook} is independent of the magnetic field, but depends on the external torque stored in the hook; a normal-size hook has a soft initial phase with $\kappa_{hook} \approx 0.4\, pN \cdot \mu m/rad$ up to ≈ π rotation and a more rigid phase thereafter [7,8]. Assuming the torsional stress in the hook results from a motor torque of approximately $1\, pN \cdot \mu m$, the hook dominates the stiffness of the system at low fields, whereas at high fields, the magnetic trap dominates. Therefore the hook stiffness cannot be neglected, as is usually done for nucleic acid tethers, and should be considered when assessing the thermal fluctuations of the bead.

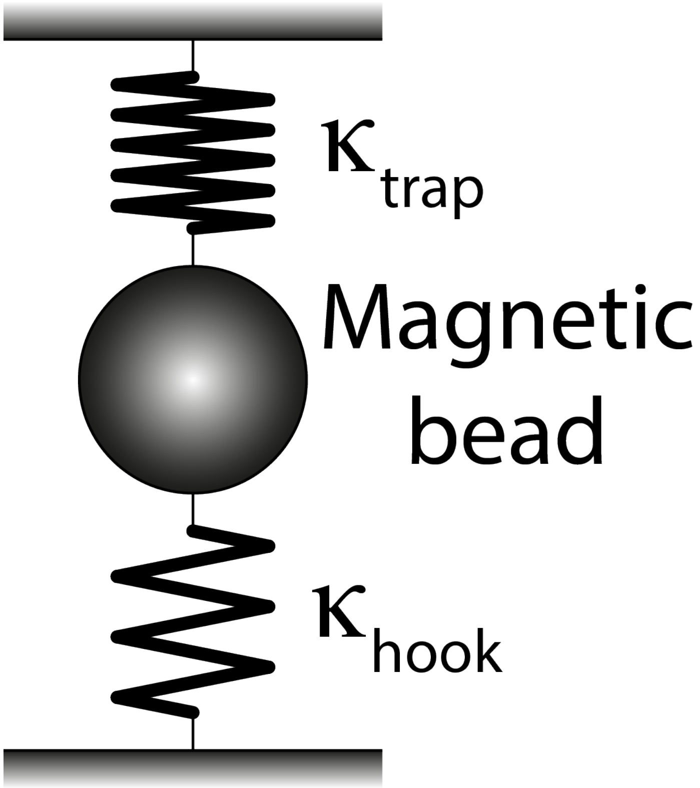

Figure S10 Double spring system. The two torsional springs, the magnetic trap and the hook, work on the magnetic bead in parallel.

S9 Instantaneous speed in the deterministic approximation

In **Supplement S2**, the third remark about the sine approximation mentions the instantaneous speed of the motor. Here, we address the instantaneous speed of the motor in the deterministic regime in contrast to the average speed of the motor covered in **Supplement S3**.

Rewriting Equation S.9 of Ref. [4] gives the angle of the bead:

$$x = \operatorname{arccot}\big(A\tan\big(B(t-t_0)\big)\big) - \pi/4 \qquad \textbf{Equation S15}$$

where $A = \sqrt{\frac{C_2+C_1}{C_2-C_1}}$, $B = \sqrt{C_2^2 - C_1^2}$, and x, C_1, and C_2 are as defined in **Supplement S3**, i.e., $x = \theta - \theta_0$, $C_1 = \tau_0/\gamma_{bead}$, and $C_2 = \tau_{motor}/\gamma_{bead}$. An example trace is plotted in **Figure S11**(A).

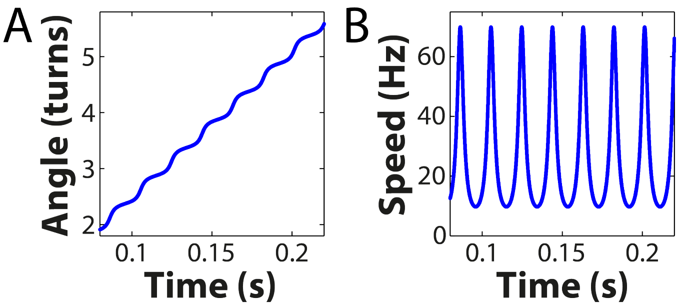

Figure S11 Angle and speed trace in the deterministic approximation. (A) The “unwrapped” angle vs. time. (B) The instantaneous speed vs. time. The traces correspond to the data in **Figure S12** at 6 mm magnet height.

The derivative of **Equation S15** gives the instantaneous speed:

$$\dot{x} = -\frac{A\,B\sec\left(B(t-t_0)\right)^2}{1 + A^2\tan\left(B(t-t_0)\right)^2} \qquad \textbf{Equation S16}$$

An example of an instantaneous speed trace is plotted in **Figure S11**(B).

At a range of magnet heights, we compute the instantaneous speed over multiple turns, and we determine the average speed, its standard deviation, its maximum, and its minimum. The results are plotted below.

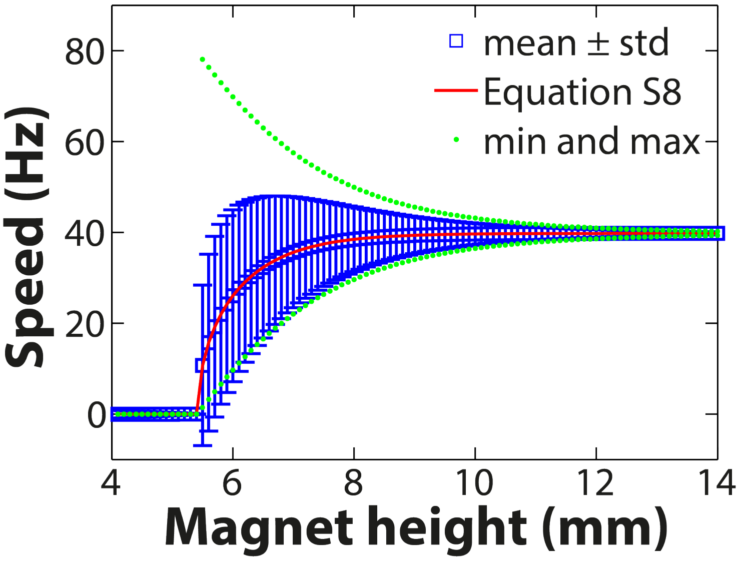

Figure S12 Speeds versus magnet height. The blue data show the average speed over multiple turns and its standard deviation. The green data show the highest and lowest speeds. The red line is the analytical expression given in **Equation S8**.

As already mentioned in **Supplement S2**, the instantaneous motor speed at certain bead orientations actually increases compared to the drag-limited speed. This happens because at those orientations the motor torque and magnetic torque work in the same direction.

Supplementary References

[1] K. Berg-Sørensen and H. Flyvbjerg, Rev. Sci. Instrum. **75**, 594 (2004).

[2] X. Chen and H. C. Berg, Biophys. J. **78**, 1036 (2000).

[3] M. M. van Oene, L. E. Dickinson, F. Pedaci, M. Köber, D. Dulin, J. Lipfert, and N. H. Dekker, Phys. Rev. Lett. **114**, 218301 (2015).

[4] F. Pedaci, Z. Huang, M. M. van Oene, S. Barland, and N. H. Dekker, Nat. Phys. **7**, 259 (2011).

[5] J. Lipfert, X. Hao, and N. H. Dekker, Biophys. J. **96**, 5040 (2009).

[6] K. Sasaki and S. Amari, J. Phys. Soc. Japan **74**, 2226 (2005).

[7] S. M. Block, D. F. Blair, and H. C. Berg, Nature **338**, 514 (1989).

[8] S. M. Block, D. F. Blair, and H. C. Berg, Cytometry **12**, 492 (1991).
